# Anticoagulant effect of prothrombin complex concentrates: a whole blood *in vitro *study

**DOI:** 10.1186/cc9851

**Published:** 2011-03-11

**Authors:** G Scharbert, U Thaler, C Weilnböck, L Wetzel, S Kozek-Langenecker

**Affiliations:** 1 Medical University Vienna, Austria; 2 Evangelical Hospital, Vienna, Austria

## Introduction

Prothrombin complex concentrates (PCCs) are currently used to treat congenital or acquired coagulation factor deficiency. PCC preparations mostly contain heparin to prevent thromboembolic events. In factor VIII and IX deficient plasma, Takeyama and colleagues observed *in vitro *a heparin effect of PCCs [1]. The aim of the present experiment was to investigate anticoagulant effects of PCCs at clinically relevant concentrations in whole blood. In an *in vitro *experiment we compared the PCC preparation used in the experiments of Takeyama and colleagues with a new heparin-free PCC preparation.

## Methods

After ethics committee approval and informed consent, citrated whole blood was obtained from 10 healthy volunteers. Two commercially available PCCs were tested: heparin-containing Prothromplex^® ^(Baxter, Austria) and heparin-free Cofact^® ^(Sanquin, the Netherlands) at concentrations of 0.3125, 0.625 and 1.25 IU/ml. Protamine was added to another set of samples (1:1 heparin:protamine). For global coagulation monitoring we used the NATEM test in the rotational thrombelastometry ROTEM^® ^(Pentapharm, Germany).

## Results

In the heparin PCC preparation we observed a significant concentration-dependent prolongation in coagulation time (CT) and coagulation formation time (CFT), even at the lowest concentration. The maximum clot firmness (MCF) was significantly reduced too. Heparin-dependent anticoagulation was reversible by protamine. The heparin-free PCCs did not affect onset of coagulation. The interpretation of the α-angle showed no increased thrombus formation in heparin-free PCC preparation.

## Conclusions

Our results confirm and extend the report of Takeyama and colleagues. At clinical relevant concentrations, PCC has an anticoagulant effect. The heparin content of PCCs should be considered in the clinical routine.
